# TIGAR Deficiency Blunts Angiotensin-II-Induced Cardiac Hypertrophy in Mice

**DOI:** 10.3390/ijms25042433

**Published:** 2024-02-19

**Authors:** Xiaochen He, Quinesha A. Williams, Aubrey C. Cantrell, Jessie Besanson, Heng Zeng, Jian-Xiong Chen

**Affiliations:** Department of Pharmacology and Toxicology, School of Medicine, University of Mississippi Medical Center, Jackson, MS 39216, USA; xhe2@umc.edu (X.H.); qawilliams@umc.edu (Q.A.W.); acantrell@umc.edu (A.C.C.); jbesanson@umc.edu (J.B.)

**Keywords:** TIGAR, cardiac remodeling, hypertrophy, glycolysis, heart failure

## Abstract

Hypertension is the key contributor to pathological cardiac hypertrophy. Growing evidence indicates that glucose metabolism plays an essential role in cardiac hypertrophy. TP53-induced glycolysis and apoptosis regulator (TIGAR) has been shown to regulate glucose metabolism in pressure overload-induced cardiac remodeling. In the present study, we investigated the role of TIGAR in cardiac remodeling during Angiotensin II (Ang-II)-induced hypertension. Wild-type (WT) and TIGAR knockout (KO) mice were infused with Angiotensin-II (Ang-II, 1 µg/kg/min) via mini-pump for four weeks. The blood pressure was similar between the WT and TIGAR KO mice. The Ang-II infusion resulted in a similar reduction of systolic function in both groups, as evidenced by the comparable decrease in LV ejection fraction and fractional shortening. The Ang-II infusion also increased the isovolumic relaxation time and myocardial performance index to the same extent in WT and TIGAR KO mice, suggesting the development of similar diastolic dysfunction. However, the knockout of TIGAR significantly attenuated hypertension-induced cardiac hypertrophy. This was associated with higher levels of fructose 2,6-bisphosphate, PFK-1, and Glut-4 in the TIGAR KO mice. Our present study suggests that TIGAR is involved in the control of glucose metabolism and glucose transporters by Ang-II and that knockout of TIGAR attenuates the development of maladaptive cardiac hypertrophy.

## 1. Introduction

Hypertension is one of the significant risk factors for heart disease and stroke, which are the leading causes of death in the United States [1]. Hypertension increases the workload of the left ventricle (LV) and induces cardiac hypertrophy as a compensatory response [2]. Clinical studies revealed that patients with LV hypertrophy are associated with a higher incidence of congestive heart failure and sudden cardiac death compared with those without hypertrophy [3], and the renin–angiotensin system is known to play a key role in cardiac hypertrophy and heart failure (HF). Angiotensin-II (Ang-II), a major effector of the renin–angiotensin system, is aberrantly activated in heart disease and plays a key role in pathological remodeling and HF [2,4]. 

Growing evidence suggests that energy metabolism is impaired or reprogrammed in cardiac hypertrophy and HF [5,6]. Ang-II has been shown to induce mitochondrial dysfunction and alter cardiac substrate use and the metabolomic profile in cardiac hypertrophy involving Sirtuin 1 (SIRT1) [2,7]. TP53-induced glycolysis and apoptosis regulator (TIGAR) has been shown to regulate mitochondrial function in fast-twitch oxidative skeletal muscle through the SIRT1-PGC1-α pathway [8]. TIGAR also plays an important role in glycolysis, which is finely regulated by 6-phosphofructo-2-kinase/fructose-2,6-bisphosphatase isoform 3 (PFKFB3). PFKFB3 produces fructose 2,6-bisphosphate (F2,6-BP), a potent allosteric activator of phosphofructokinase 1 (PFK-1) [9], which accelerates glycolysis [9,10,11]. In contrast, TIGAR breaks down F2,6-BP and inhibits glycolysis [12,13]. Okawa and colleagues revealed that the ablation of TIGAR in cardiomyocytes preserved myocardial energetics and cardiac function in a pressure overload (PO)-induced HF model [14]. Our previous studies also demonstrated that the knockout of TIGAR preserved myocardial angiogenesis, coronary vascular function, and diastolic function [15]. Hypertension leads to persistent hemodynamic overload, which induces LV hypertrophy, fibrosis, and impaired LV systolic and diastolic function, predisposing to HF. At present, the role of TIGAR in modulating glucose metabolism in hypertension-induced HF has not been studied. 

The present study examined whether TIGAR played a role in chronic Ang-II-induced hypertension, myocardial hypertrophy, and glucose metabolism by using the TIGAR knockout (TIGAR KO) mice. The results demonstrate that knockout of TIGAR in mice increased levels of F2,6-BP, upregulated PFK1, and attenuated myocardial hypertrophy but had little effect on blood pressure, cardiac dysfunction, and fibrosis. Our present data suggest a fundamental role of TIGAR in cardiac hypertrophy in Ang-II-induced hypertension.

## 2. Results

### 2.1. TIGAR Deficiency Does Not Affect Systolic Function in Ang-II-Induced Hypertension

**Figure 1:**
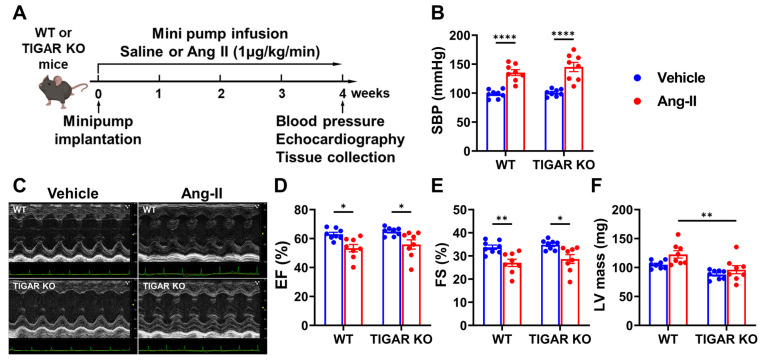
Effect of TIGAR deficiency on systolic function in Ang-II-induced hypertension. (**A**) Schematic of the experimental design. (**B**) Systolic blood pressure after four weeks of Ang-II infusion measured by the tail-cuff method. (**C**) The representative echocardiographic images of WT and TIGAR KO mice subjected to either vehicle or Ang-II infusion for four weeks. (**D**–**F**) Left ventricular (LV) ejection fraction (EF), fractional shortening (FS), and LV mass measured by echocardiography in the indicated groups (n = 8). * *p* < 0.05, ** *p* < 0.01, **** *p* < 0.0001.

Ablation of TIGAR has been shown to protect cardiac function from pressure overload-induced HF [15]. To investigate the role of TIGAR in cardiac function in Ang-II-induced hypertension, WT and TIGAR KO mice were infused with 1 µg/kg/min of Ang-II via mini-pump for four weeks. The experimental design is shown in Figure 1A. As expected, Ang-II infusion caused a significant elevation in systemic blood pressure in both WT and TIGAR KO mice when compared to their vehicle controls (Figure 1B). However, the blood pressure was not different between the two groups. Echocardiographic measurements are shown in Table 1. Ang-II infusion resulted in a similar reduction of systolic function in both WT and TIGAR KO mice, as evidenced by the comparable decrease in LV ejection fraction and fractional shortening in both groups (Figure 1C–E). Interestingly, knockout of TIGAR significantly reduced LV mass compared to the WT mice + Ang-II group (Figure 1F), suggesting that TIGAR-mediated glycolysis may play a role in Ang-II-induced cardiac hypertrophy.

### 2.2. TIGAR Deficiency Does Not Affect Diastolic Function in Ang-II-Induced Hypertension 

**Figure 2:**
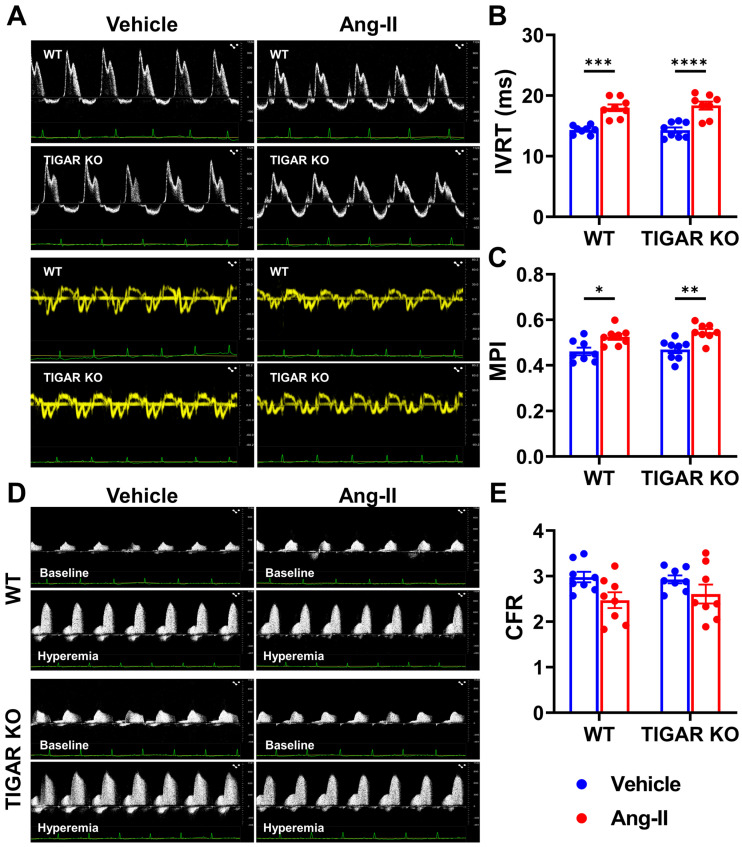
Effect of TIGAR deficiency on diastolic function in Ang-II-induced hypertension. (**A**) The representative pulsed-wave Doppler and tissue Doppler images from an apical four-chamber view of WT and TIGAR KO mice infused with either vehicle or Ang-II for four weeks. (**B**) The isovolumic relaxation time (IVRT) was increased in the WT and TIGAR KO mice after Ang-II infusion for four weeks. (**C**) The myocardial performance index (MPI) was also increased in the WT and TIGAR KO mice after Ang-II infusion for four weeks. (**D**) The representative pulsed-wave Doppler images of the proximal left coronary arteries of WT and TIGAR KO mice infused with either vehicle or Ang-II for four weeks. (**E**) The coronary flow reserve (CFR) was not affected by Ang-II infusion. n = 8, * *p* < 0.05, ** *p* < 0.01, *** *p* < 0.001, **** *p* < 0.0001.

We further examined the role of TIGAR in diastolic function in Ang-II-induced hypertension (Table 2 and Figure 2). Ang-II infusion significantly elevated isovolumic relaxation time in both WT and TIGAR KO mice (Figure 2A,B). However, there was no difference between these two groups. Similarly, the myocardial performance index was increased after Ang-II infusion, but it was not different between the WT and TIGAR KO mice (Figure 2A,C). Ang-II infusion did not alter other diastolic indexes, such as E/A and E/e’ ratio (Table 2). Coronary flow reserve (CFR) showed a trend toward a decrease in the WT mice after Ang-II infusion, although not statistically significant (Figure 2D,E and Table 3). Ang-II infusion-induced reduction of CFR in TIGAR KO mice was shown to be similar to that seen in WT mice infused with Ang-II (Figure 2D,E).

### 2.3. Knockout of TIGAR Attenuates Ang-II-Induced Cardiac Hypertrophy

**Figure 3:**
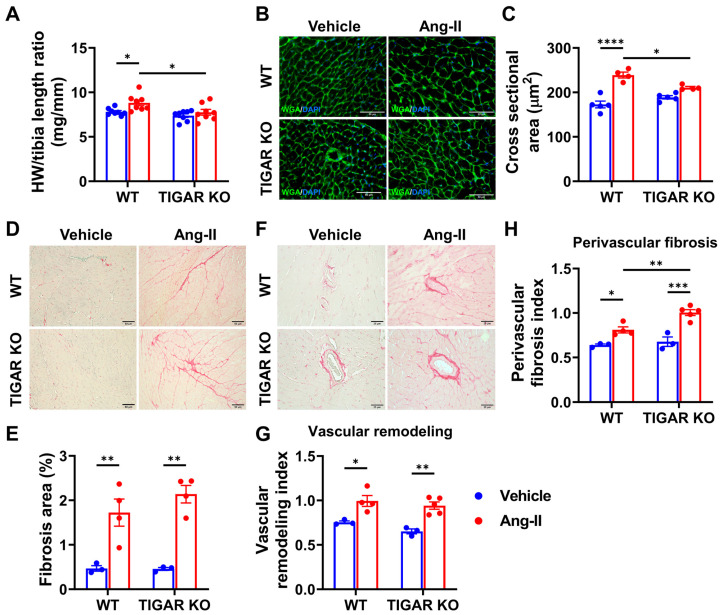
Knockout of TIGAR attenuates Ang-II-induced cardiac hypertrophy. (**A**) The ratio of heart weight to tibia length in the indicated groups (n = 8). (**B**,**C**) The representative images of wheat germ agglutinin (WGA)–stained frozen heart sections and cardiomyocyte hypertrophy were assessed by cross-sectional areas in the indicated groups (n = 4–5). A minimum of 100 cardiomyocytes from each LV section of each mouse were measured. Bar = 50 μm. (**D**,**E**) The representative images of Picrosirius red-stained paraffin-embedded heart sections and quantification of the percentage of interstitial fibrosis area in the indicated groups (n = 3–4). Bar = 50 μm. (**F**–**H**) The representative images of Picrosirius red-stained paraffin-embedded heart sections show coronary arteries and perivascular fibrosis and quantification of perivascular fibrosis index and vascular remodeling in the indicated groups. Bar = 25 μm. n = 3–4, * *p* < 0.05, ** *p* < 0.01, *** *p* < 0.001, **** *p* < 0.0001.

We further investigated the role of TIGAR in cardiac remodeling induced by volume overload. As expected, Ang-II infusion induced moderate LV hypertrophy in WT mice when compared to the vehicle control, as evidenced by a significant increase in the heart weight to tibia length ratio (Figure 3A). Moreover, wheat germ agglutinin (WGA) staining revealed a significant increase in the cross-sectional area of cardiomyocytes after Ang-II infusion in WT mice (Figure 3B,C). In contrast, deficiency of TIGAR blunted Ang-II-mediated cardiac hypertrophy. 

As shown in Figure 3D,E, Ang-II-induced interstitial fibrosis was comparable between the WT and TIGAR KO mice. Ang-II also induced similar vascular hypertrophy in WT and TIGAR KO mice (Figure 3F,G). In addition, Ang-II induced a significant increase in perivascular fibrosis in TIGAR KO mice when compared to the WT mice (Figure 3F,H). 

### 2.4. Knockout of TIGAR Increases Glycolytic Enzyme PFK-1 and Glucose Transporter Glut-4 Expression

**Figure 4:**
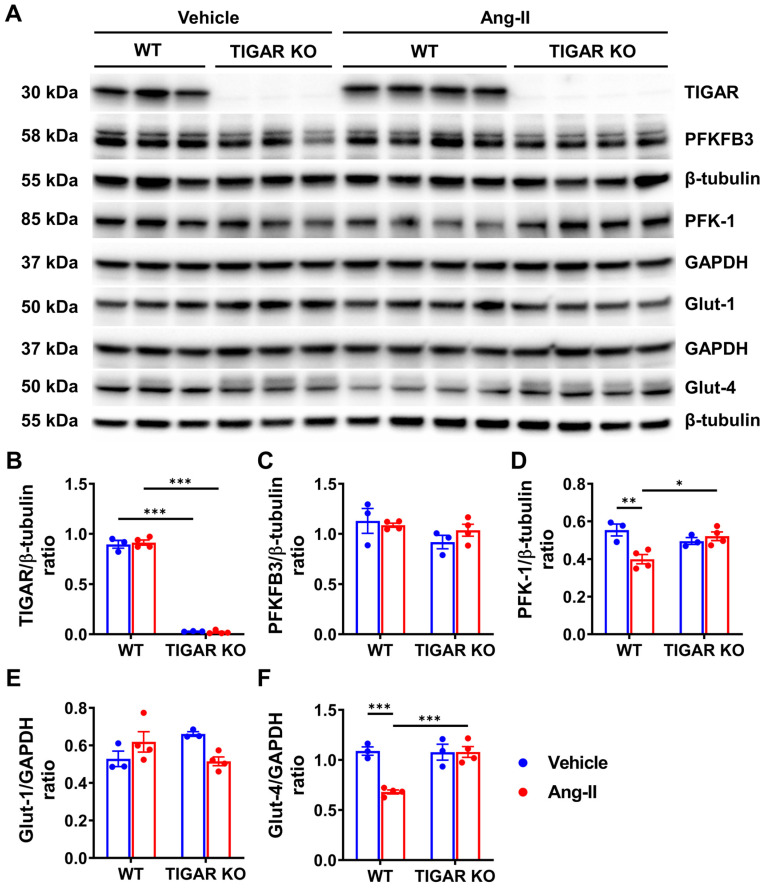
Knockout of TIGAR increases glycolytic enzyme PFK-1 and glucose transporter Glut-4. (**A**–**F**) Representative immunoblots and quantitative analysis of TIGAR, PFKFB3, PFK-1, Glut-1, Glut-4, and corresponding GAPDH or β-tubulin in the indicated groups. The expression of PFK-1 and Glut-4 decreased in the WT mice after Ang-II infusion but not in the TIGAR KO mice. n = 3–4, * *p* < 0.05, ** *p* < 0.01, *** *p* < 0.001.

**Figure 5:**
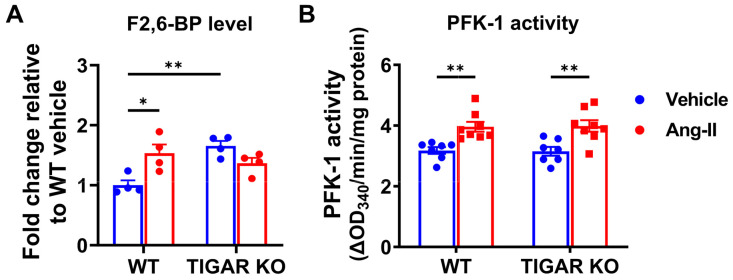
Effect of TIGAR deficiency on glycolytic enzymes in Ang-II-induced hypertension. (**A**) Cardiac F2,6-BP level was determined the coupled-enzymatic assay and expressed as the fold change to the WT sham group. n = 4. (**B**) Cardiac PFK-1 activity was determined by the coupled-enzymatic assay and expressed as the OD_340nm_/min/mg protein. n = 7–8. * *p* < 0.05, ** *p* < 0.01.

To further investigate the possible molecular mechanism by which ablation of TIGAR attenuates cardiac hypertrophy, we examined the expression of glycolysis-related proteins which are critical factors during the development of cardiac hypertrophy and HF. The expression of TIGAR was absent in the TIGAR KO mice as expected and was not changed in the WT mice after infusion with Ang-II (Figure 4A,B). The expression of PFKFB3 and Glut-1 did not change in the WT or TIGAR KO mice after Ang-II infusion (Figure 4A,C,E). Interestingly, the expression of PFK-1 and Glut-4 were significantly decreased in WT mice when compared to the TIGAR KO mice after Ang-II infusion (Figure 4A,D,F). Moreover, the coupled-enzyme method revealed that the basal levels of F2,6-BP were significantly higher in the TIGAR KO mouse hearts than in WT mouse hearts. Furthermore, levels of F2,6-BP were elevated (~1.5-fold) in the WT hearts after Ang-II infusion but did not further increase in the TIGAR KO mouse hearts (Figure 5A). PFK-1 activity was significantly increased in both WT and TIGAR KO mice after Ang-II infusion (Figure 5B).

## 3. Discussion

In the present study, we assessed the effect of TIGAR KO on cardiac metabolism, pathological hypertrophy, and cardiac dysfunction in Ang-II-induced hypertension and showed that Ang-II decreased the expression of PFK-1 and Glut-4 in association with moderate cardiac hypertrophy, which was attenuated by the ablation of TIGAR. Ang-II also induced myocardial fibrosis and impaired systolic and diastolic function, which were not affected by the ablation of TIGAR. These findings suggest that TIGAR is involved in the Ang-II-mediated alterations in cardiac metabolism and hypertrophy but has little effect on Ang-II-induced fibrosis and cardiac dysfunction.

Cardiac remodeling, including cardiac myocyte hypertrophy, perivascular, and interstitial fibrosis, is a compensatory mechanism to maintain pump function in response to hemodynamic overload such as hypertension. The renin–angiotensin–aldosterone system, primarily Ang-II via AT_1_ receptors (AT_1_-R), plays a major role in promoting hypertension, development of LV hypertrophy, and progression to HF [16]. In addition, AT_1_-R can be activated directly by mechanical stress independently of Ang-II [17]. It is therefore possible that in the Ang-II-infused animals, both hypertension and Ang-II itself can activate AT_1_-R and mediate the adaptive response. Crowley and colleagues suggested that cardiac hypertrophy depends primarily on the level of blood pressure rather than the AT_1_ receptors in the heart since heart weight and blood pressure are tightly correlated in Ang-II-induced hypertension [16]. In the present study, the blood pressure was not different between the WT and TIGAR KO mice, suggesting that TIGAR is not involved in regulating Ang-II-induced hypertension. However, our data revealed that Ang-II-induced cardiac hypertrophy was significantly reduced in TIGAR KO mice. These data suggest that TIGAR might be directly involved in the Ang-II-mediated hypertrophic response and that the beneficial effects on cardiac hypertrophy in the TIGAR KO mice were independent of blood pressure.

Emerging evidence demonstrates that cardiac hypertrophy is associated with a metabolic shift from the preferable fatty acid oxidation (FAO) to glucose oxidation [18]. Pellieux and colleagues have demonstrated that overexpression of angiotensinogen in the heart induced maladaptive cardiac hypertrophy, associated with reduction of PPARα and enzymes of fatty acid metabolism, while glucose oxidation was unchanged [19]. On the other hand, angiotensin II-induced adaptive hypertrophy of cultured cardiomyocytes is associated with decreased activation of AMP-activated protein kinase (AMPK) and increased glucose uptake, whereas AMPK activation increases fatty acid utilization and inhibits cardiomyocyte hypertrophy [20]. Therefore, the metabolic changes can be both adaptive (balanced cardiac metabolism) and maladaptive (unbalanced cardiac metabolism) to the progression of hypertrophy and may depend on the course of the disease [21]. It is therefore of interest to identify potential key regulators in cardiac remodeling involving cardiomyocyte metabolism. Our previous study demonstrated that TIGAR played a critical role in myocardial glycolytic function and that the ablation of TIGAR attenuated pressure overload-induced cardiac hypertrophy and dysfunction [22]. Previous studies also showed that reductions in F2,6-BP and PFK1 led to more profound cardiac hypertrophy during pressure overload [23,24]. 

Here, our data revealed that TIGAR KO mice had a higher basal level of F2,6-BP, which may provide early protection during Ang-II infusion. Although Ang-II infusion resulted in an elevation in the level of F2,6-BP (1.4–1.5 fold) and a small increase in PFK activity, the expression of PFK-1 was significantly decreased in the WT mice after Ang-II infusion when compared to the TIGAR KO mice. It is possible that Ang-II-induced impairment of FAO was not sufficiently compensated by an increase in glycolysis or glucose oxidation. Moreover, in the presence of TIGAR, this led to more profound cardiac hypertrophy due to preferential utilization of glucose for biomass synthesis via the pentose phosphate pathway (PPP) rather than ATP production [25]. In contrast, the knockout of TIGAR may reduce the PPP flux and thus enhance cardiac glucose utilization. Our previous study showed a very similar elevation in the level of F2,6-BP (1.4–1.5 fold) and PFK activity in TIGAR KO mice with pressure overload (PO) induced by transverse aortic constriction [22]. However, the knockout of TIGAR had little effect on improving cardiac function after Ang-II infusion. These data suggest that the heart depends more on glycolytic function during PO-induced HF and that TIGAR or glycolytic remodeling is not involved in Ang-II-induced cardiac dysfunction. Further, these differences in glycolytic metabolic remodeling and their contributions to cardiac dysfunction are most likely determined by the type, onset, and severity of heart failure. 

Glucose uptake plays a critical role in regulating cardiac hypertrophy [26,27]. Glut-1 is mainly expressed in the endothelial cells [28], whereas cardiomyocytes primarily use Glut-4 [28]. Mice with a cardiomyocyte Glut-4 deficiency developed myocardial hypertrophy and fibrosis [29]. However, the role of Ang-II and TIGAR on glucose transporters is not clear. While studies have shown that the overexpression of angiotensinogen in mice progressively decreased Glut-4 mRNA expression with unaffected Glut-1 mRNA or that Ang-II inhibited insulin-stimulated Glut-4 translocation in skeletal muscle cells [19,30], others demonstrated that Ang-II could induce membrane translocation of Glut-4 and increase glucose use in cultured cardiomyocytes [20]. Our data demonstrated that Glut-4 protein expression decreased after Ang-II infusion in WT mice, whereas the knockout of TIGAR preserved the level of Glut-4. The level of Glut-1 was not affected by Ang-II or knockout of TIGAR. These data suggest that TIGAR is involved in the control of glucose transporters by Ang-II, which may contribute to the development of maladaptive cardiac hypertrophy. 

There are limitations in the present study. First, all changes were studied at a single point, which might not be optimal for separating carbohydrate metabolism and chronic inflammation effects. Second, the present study did not evaluate the signaling pathways controlling chronic inflammation and fibrosis, usually induced in Ang II-infused animal models. We have demonstrated that TIGAR deficiency reduced endothelial inflammatory activation and attenuated TGF-β signaling and myocardial fibrosis after pressure overload-induced heart failure [22]. This study suggests that TIGAR regulates chronic inflammation and fibrotic signaling pathways, which may also be involved in the Ang II-infused animal models.

## 4. Materials and Methods

### 4.1. Mice

Male C57BL/6J mice were purchased from The Jackson Laboratory (Bar Harbor, ME) and were used as wild-type (WT) controls. Male TIGAR-deficient (TIGAR KO) mice on the C57BL/6 background were kindly gifted by Dr. Jeffrey Pessin at the Albert Einstein College of Medicine and maintained in the Laboratory Animal Facilities at the University of Mississippi Medical Center (UMMC). All animals were fed laboratory-standard chow and water and housed in individually ventilated cages. All protocols were approved by the Institutional Animal Care and Use Committee at UMMC (Protocol ID: 1564) and were in compliance with the National Institutes of Health Guide for the Care and Use of Laboratory Animals (NIH Pub. No. 85-23, Revised 1996).

Ang-II was purchased from BACHEM (#4006473, BACHEM, Torrance, CA, USA). The animal groups and experimental procedures are summarized here. The animals were randomly divided at 5–6 months of age into the following groups: WT+Vehicle, TIGAR KO+Vehicle, WT+Ang II, and TIGAR KO+Ang II. Briefly, the Alzet miniosmotic pumps (model 2001, Alzet Osmotic Pumps Company, Cupertino, CA, USA) were subcutaneously implanted under anesthesia by isoflurane. The Ang II groups were infused with 1 µg/kg/min of Ang II for four weeks (n = 8/group). The Vehicle groups were infused with sterile saline (n = 8/group). At the end of 4 weeks, blood pressure was measured by the tail-cuff method, and cardiac function was measured by echocardiography. Then, the animals were euthanized, followed by tissue collection. The hearts were rapidly excised, weighed, and snap-frozen in liquid N_2_ for further analysis.

### 4.2. Measurement of Blood Pressure

The WT and TIGAR KO mice were trained and acclimated to restraint for 20–30 min for 4 consecutive days at the same time of day for blood pressure measurement by the tail-cuff method (CODA Noninvasive Blood Pressure System, Kent Scientific, Torrington, CT, USA) without anesthesia. The final blood pressure measurement was performed on the fifth day. 

### 4.3. Echocardiography

Transthoracic echocardiograms were performed on mice using a Vevo 3100 Preclinical Imaging Platform equipped with an MX400 transducer (FUJIFILM Visual Sonics Inc., Toronto, ON, Canada). The mice were anesthetized by inhalation of 1–1.5% isoflurane mixed with 100% medical oxygen, and the heart rate was maintained between 450 and 500 beats per minute. M-mode cine loops were analyzed by Vevo LAB software (FUJIFILM Visual Sonics Inc., Canada) to measure ejection fraction (EF%), fractional shortening (FS%), and myocardial parameters, including left ventricle (LV) end-systolic diameter (LVESD), LV end-diastolic diameter (LVEDD), LV end-systolic volume (LVESV), LV end-diastolic volume (LVEDV), thickness of the anterior wall (LVAW) and posterior wall (LVPW) at end-systole and end-diastole, stroke volume (SV), and cardiac output (CO) [31]. 

Transmitral inflow pulsed-wave (PW) Doppler and Tissue Doppler (TD) imaging were used to assess diastolic function. From an apical four-chamber view, the peak velocity of early (E) and late (A) filling of mitral inflow, isovolumic relaxation time (IVRT), isovolumic contraction time (IVCT), and aortic ejection time (AET) were assessed. The myocardial performance index (MPI) was calculated using the following formula: MPI = (IVRT + IVCT)/AET. In addition, TD images were obtained from the mitral annulus to measure tissue motion velocity in early and late diastole (e’ and a’, respectively) and to calculate the E/e’ ratio [32,33,34].

CFR was assessed by PW Doppler at the left proximal coronary artery (LCA) in a modified parasternal LV short-axis view. Briefly, baseline (1% isoflurane) and hyperemic (2.5% isoflurane) coronary blood flow velocity were recorded, and the CFR was calculated as the ratio of hyperemic peak diastolic flow velocity to baseline peak diastolic flow velocity [31,33,34].

### 4.4. Tissue F2,6-BP Assay

Tissue F2,6-BP concentration was determined as previously described [35]. Briefly, samples of LV were weighed and homogenized in NaOH (0.05 M). The resulting mixture was heated for 20 min at 80 °C. After cooling, the samples were neutralized with 1 M acetic acid in the presence of 20 mM Hepes and then centrifuged. Samples were incubated at 37 °C for 5 min in the following assay mixture: 50 mM Tris, 5 mM Mg^2+^, 1 mM fructose-6-phosphate (Sigma #F3627), 0.15 mM NADH (Sigma #N4505), excessive PPi-dependent PFK-1 (enriched from potato tubers), 0.2 U/mL aldolase (Sigma #A2714), 8 U/mL triosephosphate isomerase (Sigma #T2507), and 1 U/mL glycerol-3-phosphate dehydrogenase (Sigma #10127752001). After the 5 min pre-incubation time, 0.5 mM pyrophosphate was added to start the reaction, and the rate of change in OD_340nm_ every 30 s was followed for 5 min in a Bio-Rad xMark microplate spectrophotometer (Bio-Rad, Hercules, CA, USA). Data are expressed as the fold change compared to the WT controls.

### 4.5. Phosphofructokinase Activity Assay

Tissue PFK-1 activity was determined as previously described [36]. Briefly, samples of LV were weighed and homogenized in lysis buffer, followed by sonication and centrifugation. The reaction was performed using 4 µg of total protein in a 96-well plate containing 80 µL of the reaction buffer (50 mM Tris–HCl, pH 7.5, 5 mM MgCl_2_, 5 mM ATP (Sigma #A6419), 0.2 mM NADH, 100 mM KCl, 5 mM Na_2_HPO_4_, 0.1 mM AMP (Sigma #A2252), 5 mM fructose-6-phosphate, 5 U/mL triosephosphate isomerase, 1 U/mL aldolase, and 10 U/mL glycerol-3-phosphate dehydrogenase. Absorbance at 340 nm was read at 37 °C every 30 s for a period of 30 min in a Bio-Rad xMark microplate spectrophotometer (Bio-Rad, Hercules, CA, USA). Data are expressed as the change in absorbance at 340 nm/min/mg of protein.

### 4.6. Histology and Immunofluorescence

Left ventricles were fixed with 10% neutral buffered formalin, processed, embedded in paraffin, and sectioned at 5 μm thickness. Picrosirius red staining was used to evaluate the degree of cardiac interstitial and perivascular fibrosis. A total of 5–10 fields were randomly selected for each mouse, and the fibrotic fraction was calculated as the ratio of the Picrosirius red-stained area to the total myocardial area. Fibrosis around the coronary arteries was semi-automatically quantified and expressed as perivascular fibrosis index using the following formula: perivascular fibrosis index = perivascular area (μm^2^)/vessel wall area (μm^2^). 

Cryostat sections (10 µm thickness) of the left ventricle were stained with Alexa Fluor™ 488 conjugated wheat germ agglutinin (WGA, Invitrogen, Carlsbad, CA, USA) to evaluate the size of cardiomyocyte and cardiac hypertrophy. Microscopic photos of 10 to 15 fields per section per mouse were taken using the Nikon Eclipse 80i microscope, coupled with an X-Cite^®^ 120 Fluorescence Illumination system (Nikon Instruments, Melville, NY, USA). The cross-sectional area of the cardiac myocyte was measured using ImageJ software.

### 4.7. Immunoblot Analysis

Protein extractions from heart ventricular samples were prepared in lysis buffer with a protease/phosphatase inhibitor cocktail (A32961, Thermo Fisher Scientific, Waltham, MA, USA). Lysates were separated by SDS-PAGE under reducing conditions, transferred to a PVDF membrane, and analyzed by immunoblotting. The PVDF membranes were probed with primary antibodies of Glut1, Glut4, PFKFB3, PFK1 and TIGAR (Table 4). The membranes were then washed and incubated with an anti-rabbit (31460) or anti-mouse (31430) secondary antibody conjugated with horseradish peroxidase (1:10,000, Thermo Fisher Scientific, Waltham, MA, USA). Loading controls were probed with β-actin and GAPDH antibodies. Densitometries were analyzed with Image Lab Software 6.0 (Bio-Rad, Hercules, CA, USA). 

### 4.8. Statistical Analysis

Data are presented as mean ± SD. The assumptions of normality in both comparison groups were determined by normality and long-normality tests. Statistical significance was determined using a Student’s *t*-test (two-tailed) between the means of two groups, or two-way ANOVA followed by Tukey’s post-hoc test for multiple comparisons in GraphPad Prism 8 software (San Diego, CA, USA). *p* < 0.05 was considered statistically significant.

## 5. Conclusions

Our study demonstrated a protective role of ablation of TIGAR in Ang-II-induced cardiac hypertrophy. Knockout of TIGAR has little benefit against Ang-II-induced cardiac dysfunction and fibrosis. In addition, knockout of TIGAR attenuated Ang-II-induced maladaptive cardiac hypertrophy, preserving the levels of PFK-1 and Glut-4, and increasing F2,6-BP production. This study provides new insights into the role of TIGAR in cardiac metabolism during hypertension to improve cardiac hypertrophy.

## Figures and Tables

**Table 1:** Echocardiographic measurements.

Parameters	WT		TIGAR KO	
	Vehicle (n = 8)	Ang-II (n = 8)	Vehicle (n = 8)	Ang-II (n = 8)
Heart Rate	449 ± 10	479 ± 4 *	465 ± 4	478 ± 6
Diameter; s (mm)	2.71 ± 0.09	2.57 ± 0.11	2.60 ± 0.07	2.42 ± 0.17
Diameter; d (mm)	4.00 ± 0.11	3.56 ± 0.13	3.88 ± 0.06	3.40 ± 0.15 #
Volume; s (µL)	27.65 ± 2.14	24.56 ± 2.52	24.99 ± 1.57	22.28 ± 4.60
Volume; d (µL)	70.50 ± 4.23	53.95 ± 4.73	65.48 ± 2.41	48.59 ± 5.64 #
SV (µL)	42.85 ± 2.57	29.39 ± 2.96 *	40.49 ± 1.24	26.31 ± 1.44 #
CO (mL/min)	19.17 ± 1.21	14.14 ± 1.50 *	18.84 ± 0.60	12.59 ± 0.70 #
LVAW; s (mm)	1.12 ± 0.03	1.23 ± 0.04	1.15 ± 0.03	1.11 ± 0.04
LVAW; d (mm)	0.76 ± 0.02	0.92 ± 0.03 *	0.79 ± 0.03	0.81 ± 0.03
LVPW; s (mm)	1.07 ± 0.04	1.20 ± 0.06	1.02 ± 0.03	1.07 ± 0.05
LVPW; d (mm)	0.75 ± 0.05	0.96 ± 0.06 *	0.66 ± 0.02	0.84 ± 0.04 #

Data are shown as mean ± SD. SV—stroke volume; CO—cardiac output; LVAW—left ventricular anterior wall; LVPW—left ventricular posterior wall; EF—ejection fraction; FS—fractional shortening. * *p* < 0.05 vs. WT Vehicle; # *p* < 0.05 vs. TIGAR KO Vehicle.

**Table 2:** Pulsed-wave Doppler measurement.

Diastolic Index	WT		TIGAR KO	
	Vehicle (n = 8)	Ang-II (n = 8)	Vehicle (n = 8)	Ang-II (n = 8)
E (cm/s)	84.37 ± 2.57	75.89 ± 3.12	82.28 ± 2.11	68.39 ± 3.85 #
A (cm/s)	61.58 ± 2.30	55.34 ± 3.28	61.58 ± 2.81	47.55 ± 2.87 #
E/A ratio	1.38 ± 0.06	1.41 ± 31.83	1.35 ± 0.06	1.51 ± 0.16
e’ (cm/s)	2.95 ± 0.13	2.54 ± 0.26	2.83 ± 0.05	2.20 ± 0.15 #
a’ (cm/s)	2.51 ± 0.09	2.18 ± 0.24	2.50 ± 0.06	1.75 ± 0.10 #
E/e’ ratio	29.22 ± 1.83	31.83 ± 3.10	29.16 ± 0.95	31.75 ± 2.05
IVCT (ms)	8.92 ± 0.51	8.88 ± 0.74	9.72 ± 0.52	9.28 ± 0.39
AET (ms)	49.50 ± 0.90	49.33 ± 1.52	50.38 ± 1.18	52.06 ± 2.89

Data are shown as mean ± SD. IVCT—isovolumic contraction time; AET—aortic ejection time. # *p* < 0.05 vs. TIGAR KO Vehicle.

**Table 3:** Coronary flow velocity measurement.

Coronary Flow Velocity	WT		TIGAR KO	
	Vehicle (n = 8)	Ang-II (n = 8)	Vehicle (n = 8)	Ang-II (n = 8)
S_baseline_ (cm/s)	5.67 ± 0.44	9.13 ± 1.05	6.63 ± 0.54	11.46 ± 3.65
D_baseline_ (cm/s)	28.58 ± 1.65	36.33 ± 3.19	30.59 ± 1.45	33.80 ± 3.41
S_hyperremia_ (cm/s)	25.64 ± 1.78	35.93 ± 3.41 *	28.70 ± 1.83	30.08 ± 3.19
D_hyperremia_ (cm/s)	83.73 ± 2.92	88.67 ± 4.37	82.24 ± 2.76	75.32 ± 4.37

Data are shown as mean ± SD. * *p* < 0.05 vs. WT Vehicle.

**Table 4:** Primary Antibodies for Immunoblot Analysis.

Primary Antibody	Catalog #	Host	Vender	MW (kDa)	Dilution
GAPDH	#2118	rabbit	Cell Signaling, Danvers, MA, USA	37	1:5000
Glut-1	NB110-39113	rabbit	Novus Biologicals, Centennial, CO, USA	55	1:1000
Glut-4	#2213	mouse	Cell Signaling, Danvers, MA, USA	50	1:1000
PFK-1	sc-377346	rabbit	Santa Cruz, Dallas, TX, USA	85	1:1000
PFKFB3	ab181861	rabbit	Abcam, Boston, MA, USA	58	1:1000
TIGAR	sc-67273	rabbit	Santa Cruz, Dallas, TX, USA	30	1:1000
β-tubulin	#86298	mouse	Cell Signaling, Danvers, MA, USA	55	1:2000

## Data Availability

Data are available from the authors on request.
